# Genetic parameters and allele frequencies of five new European Standard Set STR loci (D10S1248, D22S1045, D2S441, D1S1656, D12S391) in the population of Romania

**DOI:** 10.3325/cmj.2013.54.232

**Published:** 2013-06

**Authors:** Florin Stanciu, Simona Vladu, Veronica Cuţăr, Daniela Cocioabă, Florentina Iancu, Adnana Cotolea, Ionel Marius Stoian

**Affiliations:** National Forensic Science Institute, Bucharest, Romania

## Abstract

**Aim:**

To establish allele frequencies and genetic parameters for 5 new European Standard Set short tandem repeat (STR) loci in the population of Romania and to compare them with those in other populations.

**Methods:**

DNA was isolated using QIAamp 96 DNA Swab BioRobot Kit and Chelex 100 methods. Polymerase chain reaction amplification was done using Investigator ESSplexPlus Kit (D1S1656, D2S441, D2S1338, D3S1358, D8S1179, D10S1248, D12S391, D16S539, D18S51, D19S433, D21S11, D22S1045, FGA, TH01, and vWA). For DNA typing, Applied Biosystems 3500/3500xL Genetic Analyzer was used. Statistical analysis was done using Powerstats, GDA, and Arlequin software.

**Results:**

Power of discrimination and polymorphism information content was highest for two new ESS loci, D1S1656 and D12S391. Comparison of allele frequencies for 5 new ESS loci in Romanian population with previously published population data showed significant differences for all compared populations, with the exception of Hungary. Geographically more distant populations, such as Spain, Sweden, United Kingdom, Germany, and Portugal differed more than closer populations.

**Conclusion:**

New ESS STR loci are very useful for the analysis of forensic samples (persons or traces) due to their characteristics (shortness and high polymorphism). In comparisons with other common STR markers, they have a higher power of discrimination and also higher polymorphism information content, and could be used in any national DNA database.

The establishment of standard sets (or common sets) of short tandem repeat (STR) markers which had first been a necessity for the forensic scientific community, as a result of globalization became a necessity for the worldwide law enforcement agencies. STR markers standard sets facilitate communication and judicial transmission of the forensic DNA typing results between different forensic groups or countries (1).

Although several STR sets have been proposed (2), three of them are most frequently used: Interpol Standard Set of Loci – ISS (FGA, TH01, VWA, D3S1358, D8S1179, D18S51, D21S11), US Core Loci – CODIS (CSF1PO, FGA, TH01, TPOX, VWA, D3S1358, D5S818, D7S820, D8S1179, D13S317, D16S539, D18S51, D21S11), and European Standard Set of Loci – ESS (D3S1358, VWA, D8S1179, D21S11, D18S51, TH01, FGA, D1S1656, D2S441, D10S1248, D12S391, D22S1045).

The five new European Standard Set STR loci studied in Romanian population are an upgrade of an earlier version of ESS consisiting of 7 STRs (3), adopted by the European Council in 2001. The DNA Working Group of the European Network of Forensic Science Institutes (ENFSI) reviewed the usefulness of the ESS in light of the increased exchange of DNA analysis results in 2009 and recommended the expansion with 5 new ones (4).

Romania adopted the new 5 ESS loci and started to use them at the national level at the beginning of 2012 as part of the Investigator ESSplex Plus Kit, which replaced the AmpFlSTR Identifiler PCR Amplification Kit used before for Romanian National DNA Database supplying. As a consequence, and due to the lack of any study involving D10S1248, D22S1045, D2S441, D1S1656, and D12S391 loci in the Romanian population, allele distribution and genetic parameters of these loci have to be determined. The aim of this study was to establish allele frequencies and genetic parameters for 5 new ESS loci in population of Romania and to compare them with those in other populations .

## Materials and methods

### Population

This study was conducted on a population sample of 1331 unrelated persons from Romania (population of approximately 19 000 000 inhabitants) – from 31 out of a total of 41 counties (and the municipality of Bucharest) as follows: Alba (n = 51), Arad (n = 70), Argeş (n = 54), Bacău (n = 18), Bihor (n = 31), Bistriţa-Năsăud (n = 53), Botoşani (n = 94), Brăila (n = 83), Braşov (n = 30), Bucureşti (n = 40), Cluj (n = 53), Constanţa (n = 69), Dâmboviţa (n = 26), Dolj (n = 14), Galaţi (n = 24), Giurgiu (n = 8), Gorj (n = 30), Harghita (n = 39), Hunedoara (n = 37), Ialomiţa (n = 31), Iaşi (n = 29), Ilfov (n = 94), Maramureş (n = 21), Mehedinţi (n = 32), Mureş (n = 42), Prahova (n = 82), Satu-Mare (n = 9), Timiş (n = 52), Tulcea (n = 69), Vaslui (n = 28), and Vrancea (n = 18).

The samples were collected from convicted offenders, required to provide DNA samples in accordance with the 76/2008 Romanian National DNA Database law.

### DNA analysis

DNA from 1331 buccal swabs (saliva) was extracted using QIAamp 96 DNA Swab BioRobot KIT on a BioRobot Universal System (Qiagen, Hilden, Germany), according to the manufacturer’s instructions, and occasionally (when samples had to be manually processed) using Chelex 100 method (5). The human DNA obtained from collected samples was quantified using Investigator Quantiplex Kit (Qiagen) and a 7500 Fast Real-Time PCR Systems (Applied Biosystems, Foster City, CA, USA). Approximately 1 ng target DNA from each sample was amplified using the Investigator ESSplex Plus Kit (Qiagen, Hilden, Germany), according to the manufacturer’s instructions and 9600 Thermal Cyclers (Applied Biosystems). Electrophoresis, and genotyping of amplicons were carried out on the Applied Biosystems 3500/3500xL Genetic Analyzer using GeneMapper ID-X software.

### Statistical analysis

For all autosomal 15 loci of Investigator ESSplex Plus Kit, observed heterozygosity, expected heterozygosity, and *P*-values of the Hardy-Weinberg equilibrium tests were assessed using GDA version 1.1 (6) and also matching probability, power of discrimination, polymorphism information content, probability of exclusion, and typical paternity index were calculated with a modified version of Powerstats Version 1.2 (7). Population differentiation tests were carried out using allele frequencies from previously published population data for the new five ESS loci (Austria, Belgium, Croatia, Czech, Germany, Hungary, Italy, Macedonia, Poland, Portugal, Spain, Sweden, and United Kingdom) using Arlequin Software version 3.1.1 (8).

## Results

The allele frequency distributions for the 15 STR markers studied in Romanian population and statistical parameters (Hardy-Weinberg equilibrium – *P*, expected heterozygosity, observed heterozygosity, matching probability, power of discrimination, polymorphism information content, probability of exclusion, and typical paternity index) are summarized in Table 1. The two new ESS loci, D1S1656 and D12S391, had the highest power of discrimination (0.8921 and 0.8736, respectively), as well as polymorphism information content (0.8819 and 0.8604, respectively). No deviations from Hardy-Weinberg equilibrium were observed, with the exception of D3S1358 (*P* = 0.0089). After employing the Bonferroni correction for the number of loci analyzed (0.05/15 = 0.0033), the departure observed at D3S1358 locus was considered as not significant. The expected heterozygosity and the power of discrimination calculated from the allele frequencies obtained for the Romanian population revealed that in combination, the 15 STR markers have a high forensic efficiency.

**Table 1:** Allele frequencies and statistical parameters of 15 short tandem repeat (STR) loci amplified with Investigator ESSplex Plus Kit in a population sample from Romania (N = 1331)*

Allele	D1S1656	D2S441	D2S1338	D3S1358	D8S1179	D10S1248	D12S391	D16S539	D18S51	D19S433	D21S11	D22S1045	FGA	TH01	vWA
6	-	-	-	-	-	-	-	-	-	-	-	-	-	0.2588	-
7	-	-	-	-	-	-	-	-	-	-	-	-	-	0.1510	-
8	-	-	-	-	0.0173	-	-	0.0113	-	-	-	-	-	0.1289	-
9	-	0.0011	-	-	0.0094	0.0011	-	0.1326	0.0008	-	-	-	-	0.2036	-
9.3	-	-	-	-	-	-	-	-	-	-	-	-	-	0.2487	-
10	0.0041	0.1604	-	0.0004	0.0676	0.0004	-	0.0917	0.0056	-	-	0.0004	-	0.0086	-
11	0.1018	0.3298	-	0.0004	0.0939	0.0030	-	0.3035	0.0090	0.0034	-	0.1454	-	0.0004	0.0004
11.3	-	0.1071	-	-	-	-	-	-	-	-	-	-	-	-	-
12	0.1157	0.0297	-	-	0.1123	0.0233	-	0.2712	0.1165	0.1037	-	0.0105	-	-	0.0004
12.2	-	-	-	-	-	-	-	-	-	0.0041	-	-	-	-	-
12.3	-	0.0011	-	-	-	-	-	-	-	-	-	-	-	-	-
13	0.1412	0.0252	-	0.0008	0.2881	0.2077	-	0.1608	0.1337	0.2878	-	0.0034	-	-	0.0015
13.2	-	-	-	-	-	-	-	-	-	0.0120	-	-	-	-	-
13.3	-	-	-	-	-	0.0004	-	-	-	-	-	-	-	-	-
14	0.0785	0.3002	-	0.0875	0.2344	0.2919	-	0.0274	0.1698	0.3084	-	0.0763	-	-	0.1465
14.2	-	-	-	-	-	-	-	-	-	0.0316	-	-	-	-	-
14.3	0.0011	-	-	-	-	-	-	-	-	-	-	-	-	-	-
15	0.1533	0.0361	0.0015	0.2881	0.1412	0.2528	0.0222	0.0015	0.1322	0.1420	-	0.3824	-	-	0.0890
15.2	-	-	-	-	-	-	-	-	-	0.0361	-	-	-	-	-
15.3	0.0312	-	-	-	-	-	-	-	-	-	-	-	-	-	-
16	0.1360	0.0090	0.0391	0.2678	0.0331	0.1679	0.0214	-	0.1506	0.0436	-	0.2885	-	-	0.2081
16.2	-	-	-	-	-	-	-	-	-	0.0210	-	-	-	-	-
16.3	0.0342	-	-	-	-	-	-	-	-	-	-	-	-	-	-
17	0.0391	0.0004	0.1965	0.1642	0.0026	0.0492	0.0856	-	0.1473	0.0030	-	0.0793	0.0011	-	0.3002
17.2	-	-	-	-	-	-	-	-	-	0.0023	-	-	-	-	-
17.3	0.1007	-	-	-	-	-	0.0079	-	-	-	-	-	-	-	-
18	0.0026	-	0.1101	0.1792	-	0.0019	0.2085	-	0.0530	-	-	0.0094	0.0116	-	0.1781
18.2	-	-	-	-	-	-	-	-	-	0.0011	-	-	-	-	-
18.3	0.0488	-	-	-	-	-	0.0150	-	-	-	-	-	-	-	-
19	0.0004	-	0.0909	0.0116	-	0.0004	0.1540	-	0.0462	-	-	0.0045	0.0860	-	0.0635
19.3	0.0105	-	-	-	-	-	0.0075	-	-	-	-	-	-	-	-
20	-	-	0.1574	-	-	-	0.1371	-	0.0199	-	-	-	0.1206	-	0.0109
20.3	0.0008	-	-	-	-	-	0.0008	-	-	-	-	-	-	-	-
21	-	-	0.0229	-	-	-	0.1089	-	0.0098	-	-	-	0.1690	-	0.0015
21.2	-	-	-	-	-	-	-	-	-	-	-	-	0.0038	-	-
22	-	-	0.0154	-	-	-	0.1127	-	0.0038	-	-	-	0.1593	-	-
22.2	-	-	-	-	-	-	-	-	-	-	-	-	0.0162	-	-
23	-	-	0.1180	-	-	-	0.0834	-	0.0011	-	-	-	0.1529	-	-
23.2	-	-	-	-	-	-	-	-	-	-	-	-	0.0150	-	-
24	-	-	0.1251	-	-	-	0.0210	-	0.0004	-	-	-	0.1443	-	-
24.2	-	-	-	-	-	-	-	-	-	-	-	-	0.0023	-	-
25	-	-	0.1063	-	-	-	0.0113	-	0.0004	-	-	-	0.0868	-	-
25.2	-	-	-	-	-	-	-	-	-	-	-	-	0.0023	-	-
26	-	-	0.0165	-	-	-	0.0023	-	-	-	0.0038	-	0.0207	-	-
26.2	-	-	-	-	-	-	-	-	-	-	-	-	0.0015	-	-
27	-	-	0.0004	-	-	-	0.0004	-	-	-	0.0218	-	0.0053	-	-
27.2	-	-	-	-	-	-	-	-	-	-	-	-	0.0004	-	-
28	-	-	-	-	-	-	-	-	-	-	0.1356	-	0.0011	-	-
29	-	-	-	-	-	-	-	-	-	-	0.2400	-	-	-	-
29.2	-	-	-	-	-	-	-	-	-	-	0.0019	-	-	-	-
30	-	-	-	-	-	-	-	-	-	-	0.1837	-	-	-	-
30.2	-	-	-	-	-	-	-	-	-	-	0.0447	-	-	-	-
31	-	-	-	-	-	-	-	-	-	-	0.0571	-	-	-	-
31.2	-	-	-	-	-	-	-	-	-	-	0.0980	-	-	-	-
32	-	-	-	-	-	-	-	-	-	-	0.0060	-	-	-	-
32.2	-	-	-	-	-	-	-	-	-	-	0.1525	-	-	-	-
33.2	-	-	-	-	-	-	-	-	-	-	0.0466	-	-	-	-
34.2	-	-	-	-	-	-	-	-	-	-	0.0083	-	-	-	-
Ho	0.8921	0.7613	0.8731	0.7786	0.8148	0.7768	0.8736	0.7818	0.8726	0.7866	0.8496	0.7373	0.8716	0.7905	0.8016
He	0.9030	0.7550	0.8482	0.7550	0.7911	0.7881	0.8670	0.7873	0.8677	0.7798	0.8302	0.7438	0.8662	0.7776	0.7851
P	0.0708	0.3024	0.2198	**0.0089**	0.9035	0.6534	0.1933	0.6468	0.5678	0.2591	0.5746	0.6498	0.0609	0.0611	0.2950
MP	0.023	0.093	0.029	0.082	0.058	0.088	0.030	0.084	0.030	0.076	0.040	0.108	0.031	0.078	0.067
PD	0.977	0.907	0.971	0.918	0.942	0.912	0.970	0.916	0.970	0.924	0.960	0.892	0.969	0.922	0.933
PIC	0.88	0.72	0.86	0.74	0.79	0.74	0.86	0.75	0.86	0.76	0.83	0.70	0.86	0.76	0.77
PE	0.802	0.518	0.691	0.518	0.583	0.577	0.729	0.576	0.730	0.562	0.656	0.499	0.727	0.558	0.572
TPI	5.16	2.04	3.29	2.04	2.39	2.36	3.76	2.35	3.78	2.27	2.94	1.95	3.74	2.25	2.33

* Abbreviations: Ho – observed heterozygosity; He – expected heterozygosity; P-value for Hard-Weinberg equilibrium exact test based on 10 000 shufflings; MP – matching probability; PD – power of discrimination; PIC – polymorphism information content; PE – power of exclusion; TPI – typical paternity index.

Single locus comparisons with available published data for the 5 new ESS loci in other geographically close populations (Table 2) revealed significant differences between the population from Romania and other populations: at one locus with population from Austria (9), eastern part of Croatia (11), Italy (15), and southern part of Poland (17); at two loci with the population from the Czech Republic (12) and Macedonia (16); at three loci with the population from Belgium (10); at four loci with the population from Spain (19), Sweden (20), and United Kingdom (21); at five loci with the population from southern part of Germany (13) and Portugal (18).

**Table 2:** Comparison of allele frequencies on five new European Standard Set loci between Romanian population and previously published data from geographically close populations*

	Short tandem repeat loci (exact test ± standard error)				
Population	D1S1656	D2S441	D10S1248	D12S391	D22S1045
Austria (9)	**0.00026 ± 0.0002**	0.45121 ± 0.0201	0.25191 ± 0.0188	0.64064 ± 0.0282	0.12459 ± 0.0106
Belgium (10)	**0.00000 ± 0.0000**	**0.00002 ± 0.0000**	**0.00070 ± 0.0004**	0.07543 ± 0.0093	0.40804 ± 0.0178
Croatia (11)	**0.00021 ± 0.0002**	0.25187 ± 0.0181	0.49769 ± 0.0132	0.45137 ± 0.0310	0.47660 ± 0.0237
Czech Republic (12)	**0.01299 ± 0.0032**	0.06732 ± 0.0122	0.94767 ± 0.0061	**0.02511 ± 0.0055**	0.11512 ± 0.0110
Germany (13)	**0.00000 ± 0.0000**	**0.00004 ± 0.0000**	**0.00086 ± 0.0007**	**0.01921 ± 0.0049**	**0.00986 ± 0.0028**
Hungary (14)	0.09296 ± 0.0158	0.41258 ± 0.0275	0.66763 ± 0.0140	0.12622 ± 0.0074	0.76251 ± 0.0124
Italy (15)	0.14190 ± 0.0122	0.20353 ± 0.0119	0.16498 ± 0.0136	**0.02340 ± 0.0045**	0.82797 ± 0.0128
Macedonia (16)	**0.00000 ± 0.0000**	0.44990 ± 0.0252	0.67560 ± 0.0188	**0.00049 ± 0.0005**	0.87302 ± 0.0092
Poland (17)	**0.01843 ± 0.0023**	0.13526 ± 0.0152	0.35296 ± 0.0250	0.36807 ± 0.0219	0.42487 ± 0.0173
Portugal (18)	**0.00000 ± 0.0000**	**0.01463 ± 0.0054**	**0.01207 ± 0.0050**	**0.00317 ± 0.0015**	**0.00853 ± 0.0025**
Spain (19)	**0.00006 ± 0.0001**	0.71919 ± 0.0205	**0.00020 ± 0.0002**	**0.00336 ± 0.0020**	0.00734 ± 0.0024
Sweden (20)	**0.00000 ± 0.0000**	**0.01067 ± 0.0019**	**0.00085 ± 0.0007**	**0.00717 ± 0.0034**	0.15908 ± 0.0232
United Kingdom (21)	**0.00000 ± 0.0000**	**0.01884 ± 0.0059**	**0.00000 ± 0.0000**	**0.00118 ± 0.0011**	0.10185 ± 0.0128

*P value of the exact test of population differentiation. Significant differences (*P* < 0.05) are in bold.

## Discussion

For the five new 5 ESS loci, there were no significant differences between the population from Romania and Hungary (14), there was a moderate number of differences between Romania and Austria, Croatia, Italy, Poland, Macedonia, and the Czech Republic, and there was a great number of differences with the rest of the countries used for allele frequencies comparison.

Based on the number of differentiations (number of loci), it can be concluded that populations differ according to the geographic location. So, geographically distant populations, such as Spain (19), Sweden (20), United Kingdom (21), Germany (13), and Portugal (18), differed more from the Romanian population than relatively closer populations.

It seems that due to higher power of discrimination and higher polymorphism information content, these ESS markers are very good in discriminating between geographically different populations. This implies that they are very useful in human identification and therefore each country that has a national DNA database should establish their allele frequencies in order to use them for forensic purposes at the national or international level.

The analysis of the new 5 ESS STR loci extended the total number of different STR markers analyzed for Romanian population and to a certain degree confirmed the results of population comparisons done using other STR markers in four other Romanian smaller geographical and historical regions: Moldova (22), Transylvania (23), Wallachia (24), and Dobruja (25).

In conclusion, new ESS STR loci are very useful for the analysis of forensic samples (persons or traces) due to their characteristics (shortness and high polymorphism). In comparisons with other common STR markers they have a higher power of discrimination and also higher polymorphism information content. They are very helpful in daily routine of National DNA Database and should be used as a common procedure.
